# An updating approach for knowledge‐based planning models to improve plan quality and variability in volumetric‐modulated arc therapy for prostate cancer

**DOI:** 10.1002/acm2.13353

**Published:** 2021-08-02

**Authors:** Kenji Nakamura, Katsuya Okuhata, Mikoto Tamura, Masakazu Otsuka, Kazuki Kubo, Yoshihiro Ueda, Yasunori Nakamura, Kiyoshi Nakamatsu, Masao Tanooka, Hajime Monzen, Yasumasa Nishimura

**Affiliations:** ^1^ Department of Medical Physics, Graduate School of Medical Sciences Kindai University Osakasayama Japan; ^2^ Department of Radiotherapy Takarazuka City Hospital Kohama, Takarazuka Japan; ^3^ Department of Radiology Kindai University Hospital Osakasayama Japan; ^4^ Department of Radiation Oncology Osaka International Cancer Institute Chuo‐ku Japan; ^5^ Department of Radiation Oncology, Faculty of Medicine Kindai University Osakasayama Japan

**Keywords:** knowledge‐based planning, model update, RapidPlan, standardization

## Abstract

**Purpose:**

The purpose of this study was to compare the dose–volume parameters and regression scatter plots of the iteratively improved RapidPlan (RP) models, specific knowledge‐based planning (KBP) models, in volumetric‐modulated arc therapy (VMAT) for prostate cancer over three periods.

**Methods:**

A RP1 model was created from 47 clinical intensity‐modulated radiation therapy (IMRT)/VMAT plans. A RP2 model was created to exceed dosimetric goals which set as the mean values +1SD of the dose–volume parameters of RP1 (50 consecutive new clinical VMAT plans). A RP3 model was created with more strict dose constraints for organs at risks (OARs) than RP1 and RP2 models (50 consecutive anew clinical VMAT plans). Each RP model was validated against 30 validation plans (RP1, RP2, and RP3) that were not used for model configuration, and the dose–volume parameters were compared. The Cook's distances of regression scatterplots of each model were also evaluated.

**Results:**

Significant differences (*p* < 0.05) between RP1 and RP2 were found in D_mean_ (101.5% vs. 101.9%), homogeneity index (3.90 vs. 4.44), 95% isodose conformity index (1.22 vs. 1.20) for the target, V_40Gy_ (47.3% vs. 45.7%), V_60Gy_ (27.9% vs. 27.1%), V_70Gy_ (16.4% vs. 15.2%), and V_78Gy_ (0.4% vs. 0.2%) for the rectal wall, and V_40Gy_ (43.8% vs. 41.8%) and V_70Gy_ (21.3% vs. 20.5%) for the bladder wall, whereas only V_70Gy_ (15.2% vs. 15.8%) of the rectal wall differed significantly between RP2 and RP3. The proportions of cases with a Cook's distance of <1.0 (RP1, RP2, and RP3 models) were 55%, 78%, and 84% for the rectal wall, and 77%, 68%, and 76% for the bladder wall, respectively.

**Conclusions:**

The iteratively improved RP models, reflecting the clear dosimetric goals based on the RP feedback (dose–volume parameters) and more strict dose constraints for the OARs, generated superior dose–volume parameters and the regression scatterplots in the model converged. This approach could be used to standardize the inverse planning strategies.

## 1 INTRODUCTION

Compared with the conventional forward planning approach, inverse planning can improve coverage of the target and sparing of normal tissue in intensity‐modulated radiation therapy (IMRT) and volumetric‐modulated arc therapy (VMAT).^1^ However, coverage of the target and sparing of the organs at risks (OARs) with inverse planning depends on the planner's or institution's experience and protocol compliance, and can therefore compromise the gains of high‐precision radiotherapy.^2^, ^3^, ^4^, ^5^ RapidPlan (RP) which is integrated in the Eclipse treatment planning system (TPS) (Varian Medical Systems, Palo Alto, CA, USA) is a specific knowledge‐based planning (KBP) solution that can reduce the variation of dose–volume parameters and ensure planning consistency between planners and institutions.^6^, ^7^, ^8^, ^9^, ^10^, ^11^ Many studies reported that RP using a single optimization of dose–volume parameters and dose distributions can produce IMRT/VMAT plans superior or comparable to clinically accepted plans for various treatment sites.^12^, ^13^, ^14^, ^15^, ^16^, ^17^, ^18^, ^19^, ^20^, ^21^, ^22^, ^23^, ^24^, ^25^

The RP model performance, such as sparing of OARs, depends on the training plans included in the model library; therefore, the registration of better plans is useful to update the model and enhance its performance.^26^, ^27^ Wang et al. investigated the performance of a RP model updated using a closed‐loop technique and showed significant improvement in the sparing of OARs from first to second RP models.^28^ However, the problem of over‐fitting is of concern with this closed‐loop model updating technique.^28^, ^29^ Therefore, the registration of the other plans superior to the plans included in the model library can improve the performance of a RP model without causing over‐fitting.^29^ In this study, we established an original RP model and created two individual RP models by registration of the updated clinical manual plans (CMPs) for the training with two different goals. These goals were applied to improve the clinical plan's quality in a stepwise manner. Moreover, the variability of the included plans in each RP model was also evaluated as a determinant of model performance; this procedure has not been performed in past reports. The purpose of this study was to compare the dose–volume parameters and regression scatter plots of RP models created with an original optimizing method over different periods.

## 2 MATERIALS AND METHODS

### 2.1 Clinical manual plans and the model configuration process at each period

T1–T2c stage prostate cancer was examined in this study. Our institutional ethics committee approved this study. The clinical target volume (CTV) was defined as the prostate and seminal vesicle (at most 2/3 of the whole seminal vesicle) and was delineated by experienced radiation oncologists. The planning target volume (PTV) was defined with a 6‐mm posterior margin and a 10‐mm margin added to the CTV in all other directions. The OARs were rectal wall and bladder wall, which were delineated 4.0 mm inside the outer surface of the rectum and bladder. The prescribed dose was 78 Gy (39 fractions) to 95% of the volume of the PTV minus the rectum (PTV‐R).^30^

**FIGURE 1:**
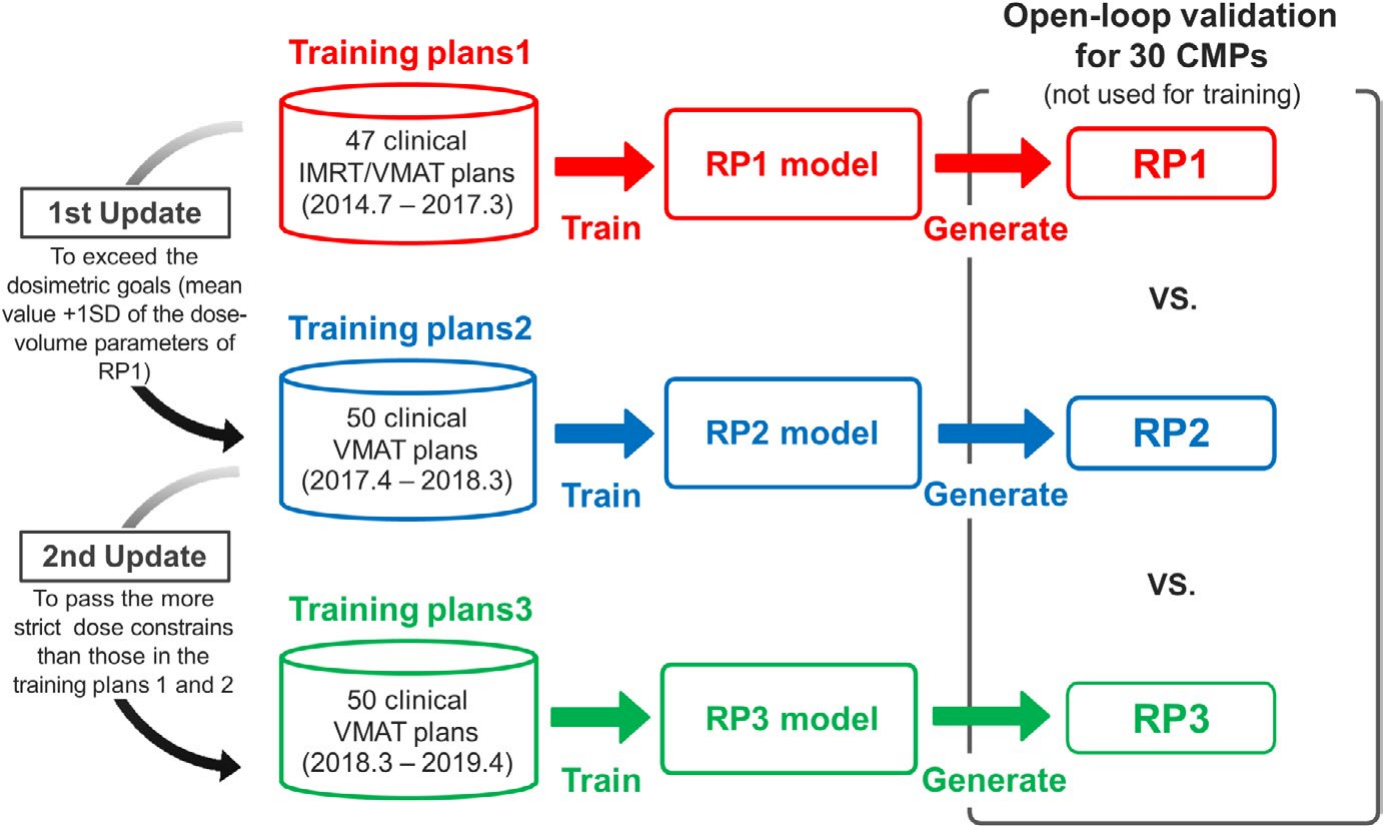
Flowchart describing the creation of RPs from the library models in a stepwise updating process over three periods. An original RP model (RP1 model) was established, and two individual RP models (RP2 and RP3 models) were created by registration of the updated different clinical manual plans for the training with the two different goals. The RP1 model was created by selecting 47 clinical IMRT/VMAT plans validated over the first period. The RP2 model was created from 50 consecutive new clinical VMAT plans created over the second period by planners who received feedback from RP1. The feedback was the superior dose–volume parameters of target and OARs of RP1, and the preferable dosimetric goals were set as the mean values +1SD of RP1. The RP3 model was created from 50 consecutive anew clinical VMAT plans validated over the third period, with the planning involving more strict dose constraints for the OARs than RP1 and 2. RP1, RP2, and RP3 were created with a single optimization using each model for the other 30 validated clinical manual plans (CMPs) that were not used for model configuration

Figure 1 presents a flowchart of the stepwise updating approach for the RP models over three periods. First, we established one original model (RP1 model) and then created two individual models (RP2 and RP3 models) using the updated different CMPs. The RP1 model was created by selecting 47 clinical IMRT/VMAT plans delivered from July 2014 to May 2017. The RP2 model was created with 50 consecutive new clinical VMAT plans delivered from April 2017 to May 2018. The CMPs for the first update were created by the planners who received feedback from the RP1 model. The feedback was the superior dose–volume parameters of target and OARs of RP1, and the preferable dosimetric goals were set as the mean values +1SD of RP1. The RP3 model was created from 50 consecutive new clinical VMAT plans delivered from May 2018 to April 2019. The CMPs for the second update used more strict dose constraints for the OARs than the RP1 and RP2 models by looking at past plans, as shown in Table 1.^30^ All CMPs were optimized to achieve the dose constraints and/or dosimetric goals by physicians and medical physicists, with no upper limit on the number of optimization rounds. All plans were checked by a single expert radiation oncologist.

**TABLE 1:** Dose constraints and preferable dosimetric goals of each structure in the three different periods. In the second period, the preferable dosimetric goals were set as the mean values +1SD of the dose–volume parameters of RP1

Structure	Parameter	Dose constraints (preferable dosimetric goals)		
		First period plan	Second period plan	Third period plan
PTV‐R	D_max_	<110%	<110%	<110%
	D_mean_	99%–103%	99%–103%	99%–103%
Rectal wall	V_40 Gy_	<60%	<60% (<51%)	<40%–50%
	V_60 Gy_	<35%	<35% (<32%)	<25%–30%
	V_70 Gy_	<25%	<25% (<19%)	<15%–20%
	V_78 Gy_	<1%	<1%	<1%
Bladder wall	V_40 Gy_	<60%	<60% (<57%)	<50%
	V_70 Gy_	<35%	<35% (<28%)	<30%

Table 2 lists the anatomical characteristics and numbers of outliers for the registered CMPs in each model library. Outliers were defined according to the model configuration statistical information using Eclipse TPS version 15.3 in the statistical analysis of the model for the rectal and bladder walls; however, outliers were not excluded from any of the models because we aimed to compare the quality and variations between the models without subjectivity.^6^, ^17^, ^31^, ^32^ All RPs (RP1, RP2, and RP3) were calculated with Eclipse version 15.3. All plans were optimized and calculated using photon optimizer (PO) and the Varian analytic anisotropic algorithm (AAA) to eliminate the dependency of the optimizer version. The validation plans were randomly selected from second and third periods and calculated with the AAA. In the RP model optimization, only line objectives were used for the OARs to eliminate any subjectivity, and the upper and lower objectives for the PTV‐R were set at 102% and 99%, respectively. All priorities were generated automatically, and the target was prioritized in the overlap region between the target and OARs. The optimization with the RP model and the calculation of the dose distributions were performed with PO and AAA version 15.3, respectively.

**TABLE 2:** Anatomical characteristics and numbers of outliers for the registered clinical manual plans in each model library

		RP1 model	RP2 model	RP3 model	Validation plans
PTV‐R volume (cm^3^)					
Mean ± SD		95.9 ± 30.8	107.4 ± 37.3	117.0 ± 30.3	111.3 ± 32.4
Maximum		205.6	218.5	235.4	193.0
Minimum		57.7	63.5	78.4	70.0
Rectal wall (cm^3^)					
Mean ± SD		20.3 ± 4.9	22.5 ± 4.6	23.0 ± 5.2	23.1 ± 4.9
Maximum		36.8	34.0	34.3	34.3
Minimum		13.4	14.3	11.0	11.0
Bladder wall (cm^3^)					
Mean ± SD		64.1 ± 29.1	52.5 ± 13.9	56.7 ± 16.1	53.0 ± 15.8
Maximum		185.8	78.6	90.8	82.9
Minimum		34.2	24.2	26.2	26.0
PTV and rectum overlap (cm^3^)					
Mean ± SD		3.4 ± 1.5	2.7 ± 1.3	3.6 ± 1.6	3.4 ± 2.0
Maximum		8.0	7.9	8.3	8.3
Minimum		1.6	0.5	0.0	0.5
Number of outliers					
Rectal wall	Geometric outliers	7	4	4	—
	Dosimetric outliers	1	0	1	—
Bladder wall	Geometric outliers	23	8	3	—
	Dosimetric outliers	0	1	0	—

### 2.2 RP VMAT plan validation

RP VMAT plans (RP1, RP2, and RP3) were created with a single optimization for 30 CMP validations (T1‐T2c prostate cancer) that were randomly selected from the second and third periods and not included in any of the model libraries (open‐loop validation), as shown in Figure 1. All VMAT plans used two full arcs (181°–179°, clockwise and counterclockwise, with collimator angles of 30° and 330°) and 10 MV photon beam. The following dose–volume parameters were compared:

1. Maximum (D_max_), minimum (D_min_), and mean (D_mean_) doses to the PTV‐R volume (D_95_ = 100%).
2. Homogeneity index (HI) = 100 × (D_2%_−D_98%_)/D_50%_, where D_98%_, D_2%_, and D_50%_ are doses received by 98%, 2%, and 50% of the PTV‐R, respectively.^15^, ^33^
3. The 95% isodose conformity index (CI_95_) = V_95%_/V_target_, where V_95%_ is the volume covered by 95% of the prescribed dose, and V_target_ is the PTV‐R volume.^6^
4. Dose–volume parameters of the rectal wall: V_40 Gy_, V_60 Gy_, V_70 Gy_, and V_78 Gy_.
5. Dose–volume parameters of the bladder wall: V_40 Gy_ and V_70 Gy_.

The differences in dose–volume parameters for each patient were compared between the models (RP1–RP2 or RP2–RP3). Table 3 shows the dose–volume parameters of the registered CMPs in each RP model.

**TABLE 3:** Mean values of dose–volume parameters for the registered clinical manual plans in each RP model library

	Mean ± SD		
	RP1 model	RP2 model	RP3 model
PTV‐R			
D_min_ (%)	88.1 ± 3.4	91.8 ± 2.4	90.6 ± 2.5
D_max_ (%)	106.7 ± 1.5	106.3 ± 1.0	106.9 ± 1.2
D_mean_ (%)	102.8 ± 0.5	102.3 ± 0.5	102.4 ± 0.6
HI	6.40 ± 0.96	5.30 ± 1.02	5.61 ± 1.11
CI_95%_	1.36 ± 0.09	1.25 ± 0.08	1.24 ± 0.05
Rectal wall			
V_40 Gy_ (%)	47.5 ± 4.4	46.6 ± 6.8	47.3 ± 5.3
V_60 Gy_ (%)	27.3 ± 3.4	26.7 ± 4.6	28.6 ± 3.5
V_70 Gy_ (%)	15.8 ± 2.5	16.3 ± 4.2	18.3 ± 3.0
V_78 Gy_ (%)	0.2 ± 0.4	0.1 ± 0.2	0.2 ± 0.3
Bladder wall			
V_40 Gy_ (%)	40.0 ± 11.0	42.8 ± 12.2	43.1 ± 9.8
V_70 Gy_ (%)	20.9 ± 5.2	20.6 ± 6.0	22.4 ± 5.4

### 2.3 Variation in the regression scatterplots of each model

RP performs principal component analysis of the actual dose–volume histogram (DVH) and geometry‐based expected dose–volume histogram (GEDVH), and the resulting correlation is used for the dose prediction of the OARs.^34^ We analyzed the correlations of the first principal component scores (PCSs1) between the DVH and GEDVH for the three models based on the model analytics tool. The resulting scatterplots and regression equations were evaluated, and variations in the scatterplots of each model were compared using *R* ^2^, Cook's distance and mean squared error (MSE).^34^, ^35^, ^36^ *R* ^2^ represents the coefficient for the determination of regression model parameters.^35^ Cook's distance is calculated by removing one plan data from the model and recalculating the regression. It summarizes how much all of the values in the regression model change when its plan data are removed. Cook's distance indicates the influential data points in a regression model, and a high Cook's distance value can have a significant negative effect on the dose prediction according to the regression line.^34^ The MSE is defined as the expected value of the square of the difference between the original and the estimated data.^37^ The MSE describes the capability of model estimation for the original DVH in a training plan, and scores closer to zero indicate a better estimation of the model for plans that are not part of the library.^35^

### 2.4 Statistical analysis

Significant differences in dose–volume parameters for comparisons of the CMPs versus each RP, RP1 versus RP2, and RP2 versus RP3, were evaluated using the Wilcoxon signed‐rank test. All statistical analyses were performed using R version 3.4.2 (The R Foundation for Statistical Computing), and *p* < 0.05 was considered to indicate statistical significance.

## 3 RESULTS

### 3.1 RP VMAT plan verification

Table 4 lists the average dose–volume parameters of the CMPs and RPs over the three periods. Comparison of the dose–volume parameters between each RP reveals that the conformities of the PTV‐R and dose–volume parameters of all OARs in RP2 were significantly superior to those in RP1, although the homogeneity of the PTV‐R in RP2 was inferior to that in RP1. On the other hand, there was significant difference in only the V_70 Gy_ of the rectal wall between RP2 and RP3. At all periods, in comparison with the CMPs, the dose–volume parameters of the RPs were superior for the PTV‐Rs and superior or comparable for the OARs, except for the V_78 Gy_ of the rectal wall.

**TABLE 4:** Comparison of average dose–volume parameters of the CMPs and RPs over the three periods

	Mean ± SD				*p*‐value				
	CMPs	RP1	RP2	RP3	CMPs versus RP1	CMPs versus RP2	CMPs versus RP3	RP1 versus RP2	RP2 versus RP3
PTV‐R									
D_min_ (%)	91.6 ± 2.1	92.5 ± 1.6	92.8 ± 1.5	92.6 ± 1.8	0.02	<0.01	0.01	0.13	0.22
D_max_ (%)	106.8 ± 1.2	105.2 ± 0.8	105.4 ± 0.6	105.6 ± 0.8	<0.01	<0.01	<0.01	0.19	0.06
D_mean_ (%)	102.5 ± 0.6	101.5 ± 0.4	101.9 ± 0.3	101.8 ± 0.3	<0.01	<0.01	<0.01	<0.01	0.33
HI	5.62 ± 1.15	3.90 ± 0.41	4.44 ± 0.36	4.41 ± 0.36	<0.01	<0.01	<0.01	<0.01	0.86
CI_95%_	1.24 ± 0.05	1.22 ± 0.04	1.20 ± 0.03	1.20 ± 0.03	<0.01	<0.01	<0.01	<0.01	0.38
Rectal wall									
V_40 Gy_ (%)	48.1 ± 5.4	47.3 ± 3.4	45.7 ± 4.3	45.2 ± 4.4	0.16	<0.01	<0.01	<0.01	0.53
V_60 Gy_ (%)	28.2 ± 4.4	27.9 ± 3.6	27.1 ± 3.8	27.6 ± 3.9	0.51	0.02	0.09	0.02	0.27
V_70 Gy_ (%)	17.9 ± 4.4	16.4 ± 2.7	15.2 ± 2.5	15.8 ± 2.4	0.05	<0.01	0.01	<0.01	<0.01
V_78 Gy_ (%)	0.1 ± 0.3	0.4 ± 0.5	0.2 ± 0.2	0.3 ± 0.2	<0.01	0.12	0.02	0.02	0.06
Bladder wall									
V_40 Gy_ (%)	44.7 ± 12.3	43.8 ± 12.8	41.8 ± 11.1	42.5 ± 12.2	0.03	<0.01	<0.01	<0.01	0.82
V_70 Gy_ (%)	21.5 ± 6.4	21.3 ± 6.6	20.5 ± 6.1	20.9 ± 6.2	0.92	<0.01	<0.01	<0.01	0.72

**FIGURE 2:**
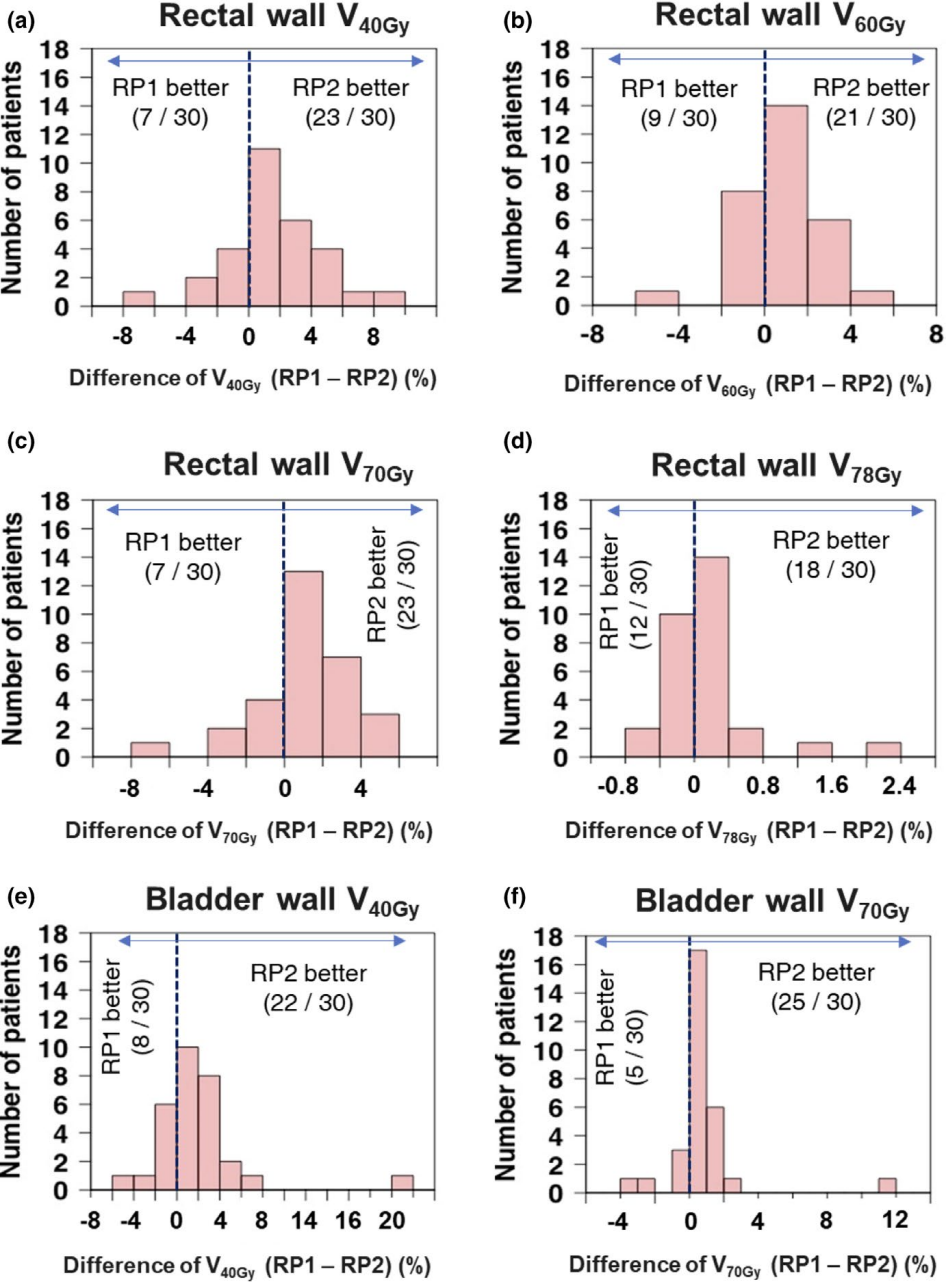
Histograms of the differences from RP1 to RP2 for the V_40 Gy_ (a), V_60 Gy_ (b)_,_ V_70 Gy_ (c), and V_78 Gy_ (d) of the rectal wall and V_40 Gy_ (e) and V_70 Gy_ (f) of the bladder wall

Figure 2 shows histograms of the differences (RP1–RP2) of dose–volume parameters for the rectal and bladder walls. The rectal and bladder walls in RP2 dose–volume parameters were lower than those of RP1 in more than 60% and 70% of cases, respectively.

### 3.2 Variation of regression scatterplots in each model

**FIGURE 3:**
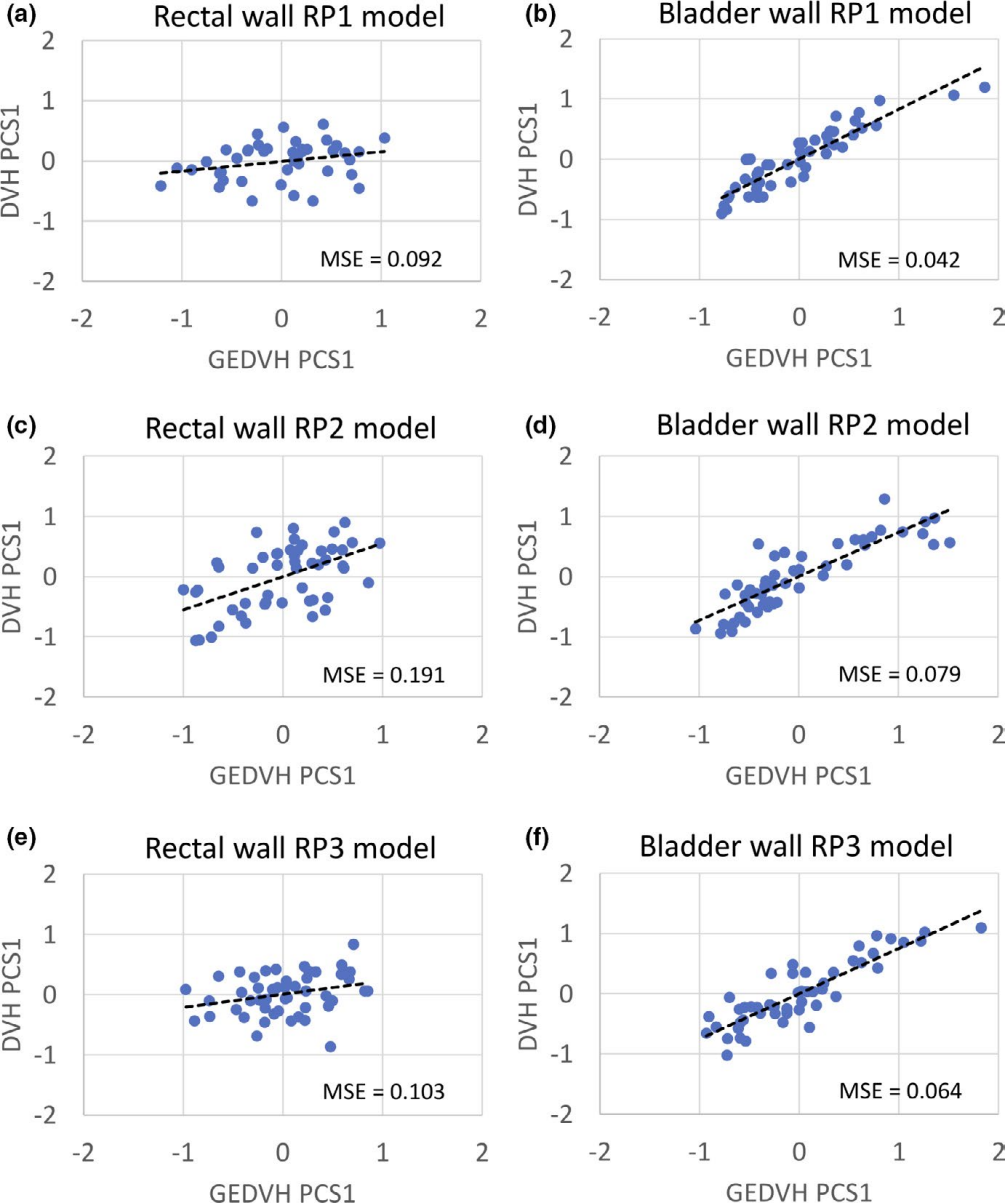
Scatterplots and regression lines of the first principal component scores (PCSs 1) of the dose–volume histogram (DVH) and geometry‐based expected dose–volume histogram (GEDVH) for the rectal wall and bladder wall in the RP1 (a, b), RP2 (c, d), and RP3 models (e, f)

**FIGURE 4:**
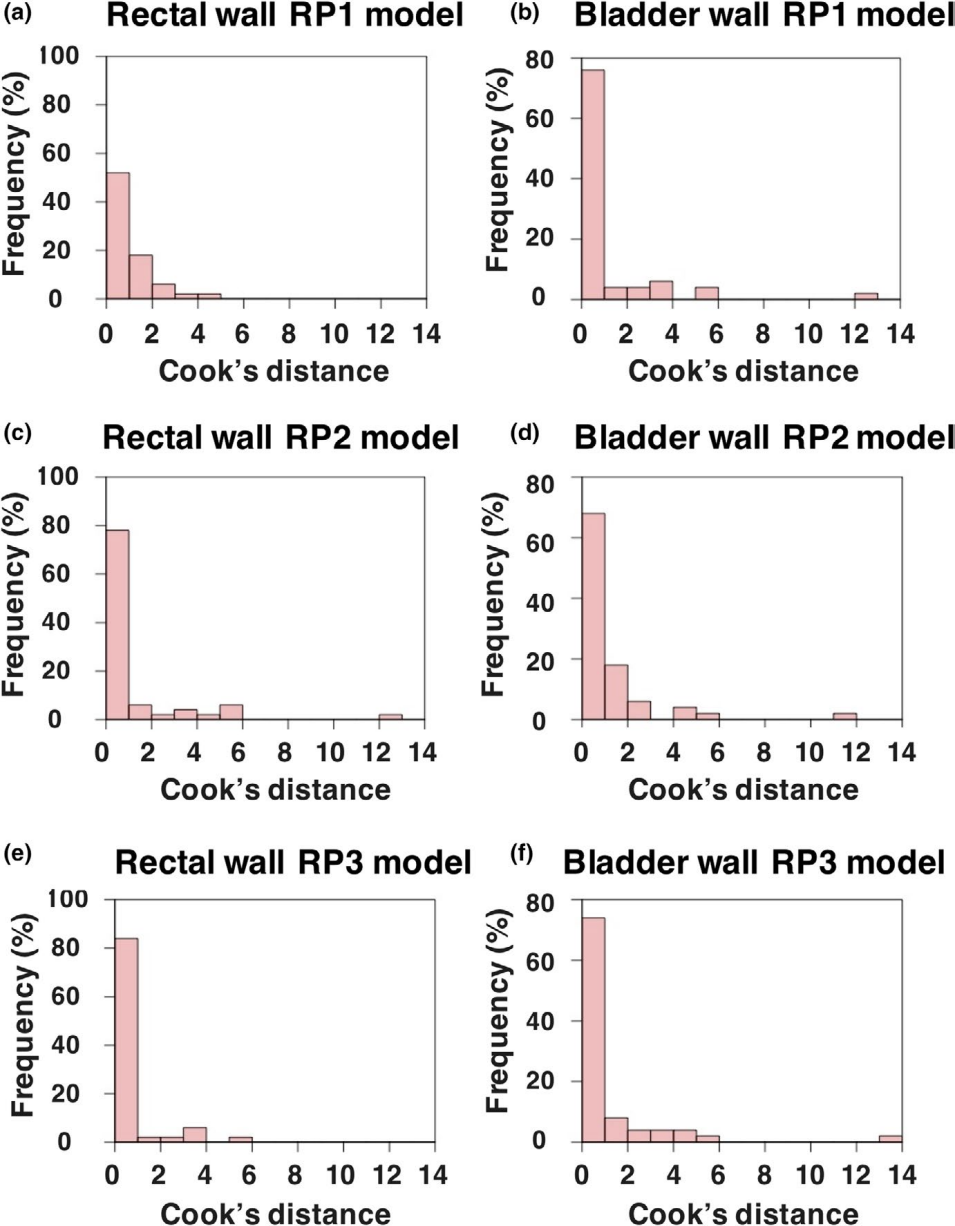
Histograms of Cook's distance for the regression line in the rectal wall and bladder wall in the RP1 (a, b), RP2 (c, d), and RP3 models (e, f)

Figure 3 shows scatterplots and regression lines for comparisons of PCSs1 between DVHs and GEDVHs in the rectal and bladder walls, and Figure 4 shows histograms of the Cook's distances for the scatterplots in Figure 3. The *R* ^2^ in the RP1, RP2, and RP3 models were 0.208, 0.550, and 0.375 for the rectal wall, and 0.841, 0.779, and 0.797 for the bladder wall, respectively. The MSEs in the RP1, RP2, and RP3 models were 0.092, 0.191, and 0.103 for the rectal wall, and 0.042, 0.079, and 0.064 for the bladder wall, respectively (Figure 3). The proportions of cases with a Cook's distance of <1.0 in models of RP1, RP2, and RP3 were 55% (26/47), 78% (39/50), and 84% (42/50) for the rectal wall, and 77% (36/47), 68% (34/50), and 76% (38/50) for the bladder wall, respectively (Figure 4).

## 4 DISCUSSION

In this study, three RP models were updated in a stepwise manner using manually optimized clinical plans that were not duplicated for each model to avoid over‐fitting. Our original updating approach involving the registered training plans with feedback from the RPs and more strict dose constraints improved the RP quality in respect to both better sparing of OARs maintaining the target coverage as shown by the values in Table 4 and the low variation in the regression scatterplots with an increase in the proportion with a Cook's distance of <1.0 and low MSE as shown in Figures 3 and 4.

For the registered plans, the homogeneity and conformity of the PTV‐R and the sparing of OARs in models RP2 and RP3 were superior or comparable to those in RP1, respectively, as shown in Table 3. On the other hand, all dose–volume parameters except for the homogeneity of the PTV‐R were significantly superior in RP2 and RP3 than in RP1. It is believed that the reasons why the homogeneity of the PTV‐R was improved in the RP1 model was to prioritize the manual upper and lower objectives for PTV‐R in the optimization process, and it caused lower sparing of the OARs due to the trade‐offs between the target and OARs compared with RP2 and RP3 models. The dose–volume parameters of the RP tend to be better when the registered plans are better,^18^, ^26^ and our previous report revealed that the RP’s feedback led to refinement of the quality of the registered plans by multi‐institution study.^27^ However, the sparing of OARs by the model update might have an upper bound for improvement, as shown by comparison of the dose–volume parameters between RP2 and RP3 in Table 4. The dose reduction of the OARs is difficult where the overlap volume between the target and OARs is large, as described by Moore et al.^38^ The anatomical characteristics of the training plans in the RP3 model, such as the large overlap volume between the target and rectum compared with the RP2 model in Table 2, may make it difficult for the RP3 model to reduce the dose of the OARs, as shown in Table 3. It can generate the lower line objectives for OARs estimated by the RP2 model than the RP3 model.

Wang et al. compared dose–volume parameters between first, second, and third RP models that were updated using a closed‐loop technique.^28^ In their study, the manual plans that were used to configure the first model were reoptimized with the model (first closed‐loop) and a second model was created from these reoptimized plans. Then, these new plans were reoptimized again with the second model (second closed‐loop) and a third model was created. They showed that sparing of the OARs was significantly improved from the first to second models, but that there was no difference from the second to third models (as in our results) because the improvement rate of the plan quality decreased and the multiple OARs were not simultaneously improved due to trade‐offs in dose between each OAR.^28^ Additionally, their model updating approach with a closed‐loop technique can cause an over‐fitting problem.^28^, ^29^ Because of patient‐specific adjustments in the objectives, the dose–volume parameters in an open‐loop validation were not drastically different from the first to second models compared with the differences in closed‐loop validations.^28^ In our study, the dose–volume parameters were significantly improved by the updated RP models from the first to second or third models in an open‐loop validation, which indicates that over‐fitting can be avoided. Each updated model in our study had sufficient stability and robustness like some studies adopted the open‐loop validation,^6^, ^11^, ^31^ because the quality of the plans generated for new patients (which strongly depends on the quality and robustness of the plans in the library) was superior to the quality of the clinical manual plans.^6^

The proportion of the plans with a high Cook's distance value in the regression scatterplots can indicate large variations in dose prediction with the RP model, leading to large variability in the RP quality. Therefore, the uncertainty of dose prediction can be reduced by increasing the number of plans with a relatively low Cook's distance. In the rectal wall, the proportion with a Cook's distance of <1.0 gradually increased from RP1 to RP3, as shown in Figure 4, indicating a reduction in the influential data points and reducing the uncertainty of the dose prediction. Moreover, the MSEs in the RP1, RP2, and RP3 models were 0.092, 0.191, and 0.103 for the rectal wall, respectively. The RP3 model had many registered plans with a low Cook's distance and a lower or comparable MSE for the regression model compared with the prior two models. Thus, the influential data points and variation of the RP3 regression scatter plots were reduced. Therefore, compared with the RP1 and RP2 models, the RP3 model could make better estimation of the DVH in the model for plans that were not part of the training set. A function of the RP as a training tool for the planners^27^ and more strict dose constraints could standardize the planners’ strategies in the optimization process. This convergence of the plan quality in the model can improve the RP prediction accuracy and reproducibility of the registered plans^39^, also reducing the intra‐center variability in the RP quality.

## 5 CONCLUSION

The RP models, which were optimized in a stepwise manner by the clinical manual plans reflecting clear dosimetric goals based on the RP feedback (dose–volume parameters) and more strict dose constraints for the OARs, generated superior dose–volume parameters for both the target and OARs to those in previous model and the regression scatterplots in the model converged. This approach could be used to standardize the inverse planning strategies.

## 6 ETHICAL APPROVAL STATEMENT

Our institutional ethics committee approved this study (Kindai University review board number: 29–133).

## CONFLICT OF INTEREST

The authors declare that they have no conflict of interest.

## AUTHOR CONTRIBUTIONS

K Nakamura, H Monzen, and M Tamura: conceptualization. H Monzen: funding acquisition. K Nakamura, K Okuhata, and M Tamura: methodology. K Nakamura, M Tamura, M Otsuka, Y Ueda, and Y Nakamura: investigation. K Nakamura, M Tamura, K Okuhata, K Kubo, and Y Ueda: writing original draft. K Nakamura, K Okuhata, H Monzen, and M Tamura: data curation. H Monzen, M Tamura, K Nakamatsu, M Tanooka, and Y Nishimura: writing‐review & editing.

## Data Availability

The data that support the findings of this study are available on request from the corresponding author. The data are not publicly available due to privacy or ethical restrictions.
